# PPAR**γ** and MEK Interactions in Cancer

**DOI:** 10.1155/2008/309469

**Published:** 2008-06-26

**Authors:** Elke Burgermeister, Rony Seger

**Affiliations:** ^1^Department of Medicine II, Klinikum Rechts der Isar, Technical University, 81675 Munich, Germany; ^2^Department of Biological Regulation, The Weizmann Institute of Science, Rehovot 76100, Israel

## Abstract

Peroxisome proliferator-activated receptor-gamma (PPAR*γ*) exerts multiple functions in determination of cell fate, tissue metabolism, and host immunity. Two synthetic PPAR*γ* ligands (rosiglitazone and pioglitazone) were approved for the therapy of type-2 diabetes mellitus and are expected to serve as novel cures for inflammatory diseases and cancer. However, PPAR*γ* and its ligands exhibit a janus-face behaviour as tumor modulators in various systems, resulting in either tumor suppression or tumor promotion. This may be in part due to signaling crosstalk to the mitogen-activated protein kinase (MAPK) cascades. The genomic activity of PPAR*γ* is modulated, in addition to ligand binding, by phosphorylation of a serine residue by MAPKs, such as extracellular signal-regulated protein kinases-1/2 (ERK-1/2), or by nucleocytoplasmic compartmentalization through the ERK activators MAPK kinases-1/2 (MEK-1/2). PPAR*γ* ligands themselves activate the ERK cascade through nongenomic and often PPAR*γ*-independent signaling. In the current review, we discuss the molecular mechanisms and physiological implications of the crosstalk of PPAR*γ* with MEK-ERK signaling and its potential as a novel drug target for cancer therapy in patients.

## 1. INTRODUCTION

### 1.1. The janus-face of PPAR*γ*: tumor suppressor *versus* tumor promoter actions

The metabolic and cell fate regulatory functions of PPAR*γ* place this nuclear receptor (NR) [1, 2] at the cross-road of life style and diabetic comorbidity risks, which are assumed to result from the diet and/or chronic inflammation-induced sequence of preneoplastic lesions towards manifested cancer [3]. Since decades, the association of aberrant insulin signaling in diabetics and increased cancer risk has been stated, and recently validated in patient studies with respect to colon, pancreas, breast, endometrium, prostate, liver, and bladder (see, e.g., [4–7]). Although PPAR*γ* plays an important part in the transmission of insulin responses and physiological diet, little direct evidence exists relating these factors to PPAR*γ* activation and the risks of the development of cancer [6–8]. One of the reasons for the lack of knowledge on the role of PPAR*γ* is that a *bona fide* high-affinity natural ligand(s) for PPAR*γ* has not been identified yet [2].

PPAR*γ* can be activated by low-affinity ligands such as unsaturated long-chain fatty acids derived from nutrient uptake (e.g., linoleic acid) and/or inflammatory reactions (e.g., 15-deoxy-Δ(12,14)-prostaglandin J2) [9, 10]. However, those do not induce the full activity of PPAR*γ* in most systems examined [2]. As of today, modulation of PPAR*γ* activity is mediated by synthetics drugs, and among them the thiazolidinediones (TZDs) rosi- and pioglitazone are considered to be potent and selective PPAR*γ* agonists [2]. These drugs were approved as insulin sensitizers for the treatment of type-2 diabetes mellitus [11] and have been proven helpful in vascular and atherogenic complications [12, 13]. However, TZD drugs can also exert protumorigenic actions in certain rodent models [14, 15]. In addition, the safety of the TZDs has been recently evaluated in clinical studies aimed to examine cancer prevalence in diabetic patients under TZD use [16–18]. One study stated a significant association of cancer risk in women under any TZD treatment (1003 patients) [17], while the other two stated no significant associations (126,971 patients [16]; 87,678 patients [18]). On the other hand, patients with long-term intake of nonsteroidal anti-inflammatory drugs (NSAIDs), cyclooxygenase (COX) inhibitors that prevent endogenous eicosanoid production and may act also as low-affinity PPAR*γ* ligands, were reported to profit from a reduced risk for colon cancer formation [19].

These paradoxical effects resulting from PPAR*γ* activation are derived from a complex balance of anti-*versus* protumor functions of PPAR*γ* protein and its ligands in a given system. The latter are also related to the interaction of PPAR*γ* with other oncomodulating proteins (such as MEK1 and *β*catenin). In the current review, we will discuss this janus-faced role of PPAR*γ* and its ligands in cancer with a major focus on its crosstalk with the ERK signaling cascade, which is a central signaling pathway deregulated in a majority of tumor types in humans.

### 1.2. The ERK cascade and cancer

The MAPK cascades are central signaling pathways that mediate the response of essentially all cellular processes stimulated by extracellular ligand, including proliferation, survival, differentiation, apoptosis, stress response, and even oncogenic transformation. Four main cascades have been identified to date, of which the Ras-Raf-MEK1/MEK2-ERK1/ERK2 cascade (ERK cascade) is the most prominent one in human cancers [20, 21]. Its multilevel organisation of kinases guarantees signal amplification and coherence, and its scaffold proteins [22] organize the pathway into a 3D module that enables crosstalk and direct interactions with other central signaling pathways such as the PPAR*γ*s.

Within the MAPK family, the ERK cascade constitutes a major signaling pathway, regulating cell proliferation and survival, as well as cell adhesion and motility, differentiation, embryonal development, and neuronal regulation [21, 23]. Its deregulation, mainly due to constitutive upregulation by receptor kinase “gain of function” mutations, contributes to cancer initiation and progression [24–26]. The majority of human carcinomas harbour increased expression or activating point mutations for the upstream components of the ERK cascade (e.g., epidermal growth factor receptor (EGFR/Her1), Her2/Neu/ErbB2, K-Ras, B-Raf) that culminate in a higher ERK activity in a large majority of human tumors. The ERK cascade currently represents the main targeted cascade (next to the angiogenic vascular endothelial growth factor/receptor (VEGF/R) system) by second-generation low molecular weight (LMW) kinase inhibitors (e.g., gefitinib, erlotinib) and monoclonal (humanized) mAbs directed against members of the EGFR family (e.g., herceptin), which are in clinical use against cancer (as reviewed in [25, 27, 28]). Therefore, inhibitors of the ERK cascade are likely to be beneficial in combating most types of cancer.

## 2. MECHANISMS OF CROSSTALK BETWEEN PPAR*γ* AND THE ERK CASCADE

**Figure 1:**
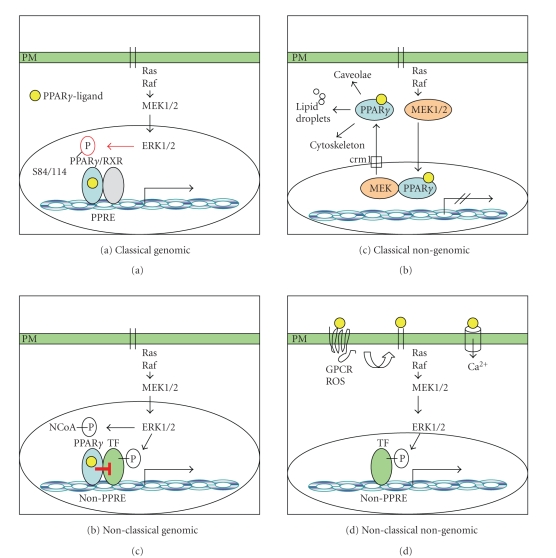
*Mechanisms of PPAR*γ*-ERK signaling crosstalk*: (a) serine phosphorylation of PPAR*γ* by the ERK cascade suppresses the classical genomic action of RXR/PPAR*γ* heterodimers on PPREs in the DNA; (b) ERK cascade phosphorylation of promitotic and proinflammatory transcription factors (TF) and NR coactivators (NCoA) modulates their interaction with PPAR*γ* “On-DNA”; (c) nuclear export of PPAR*γ* by MEK1 may result in “Off-DNA” interactions of PPAR*γ* with alternative protein partners in the cytoplasm; (d) PPAR*γ*-independent ERK cascade activation by PPAR*γ* ligands through plasma membrane GPCRs, transactivation of the EGFR (black bars), or calcium signaling.

The mechanism of action and the regulation of PPAR*γ* have attracted considerable attention over the years. Although this protein was initially shown to act as a transcription factor, studies using synthetic ligands suggested that it may exert its function via activation of signaling as well [1, 2]. According to the current knowledge, PPAR*γ* signaling is mediated by several distinct mechanisms (Figure 1). The best known one is exerted by PPAR*γ* protein itself, which is activated by ligand binding, heterodimerizes with the retinoic X receptor (RXR) and requires NR coregulator recruitment, events that lead to binding and transcriptional activation of PPAR-responsive elements (PPREs) in the DNA [29] (Figure 1(a)). Simultaneous activation of the ERK cascade (e.g., by mitogens) therein contributes to inhibition of this classical genomic action through serine phosphorylation of PPAR*γ* (Figure 1(a)). Another mechanism is that PPAR*γ* interacts with other transcription factors at the DNA level, which leads to PPRE-independent genomic actions of PPAR*γ* protein and its ligands [9, 10] (Figure 1(c)). Activation of the ERK cascade participates in this mechanism by phosphorylation of the latter transcription factors that interact with PPAR*γ* (Figure 1(c)). A third possibility is that nuclear export and cytoplasmic retention of PPAR*γ* by MEK1 [30] results in “off-DNA”-interaction of PPAR*γ* with distinct protein partners (e.g., cytoskeleton, lipid droplets, kinases), leading to alternative cytoplasmic signaling (Figure 1(b)). Finally, PPAR*γ* ligands can function via activation of intracellular signalling (e.g., the ERK cascade) by a PPAR*γ*-independent mechanism, which is derived from exogenous application of ligands that bind to plasma membrane-bound receptors [31] (Figure 1(d)). The latter mode of action can be “nongenomic,” that is, involving cytosolic signaling cascades, or “genomic,” that is, converging on the DNA by activation of alternative (non-PPAR) transcription factors (Figure 1(d)).

As apparent from the above description, interaction with the ERK cascade plays an important role in the regulation and signal transmission of PPAR*γ* and its ligands. Overall, three main mechanisms of signaling crosstalk between the ERK cascade and PPAR*γ* were described so far as follows: (1) phosphorylation of PPAR*γ* (and its cofactors) by ERKs and other MAPKs (p38, JNK); (2) nongenomic activation of the ERK cascade by PPAR*γ* ligands; and (3) compartmentalization of PPAR*γ* by the ERK cascade component MEK1. Those are described in details in this section.

### 2.1. The functions of the PPAR*γ* protein and its regulation by ERK phosphorylation

Genetic and pharmacologic studies in cells, rodent models, and human patients corroborated that the PPAR*γ* protein serves as a master regulator of adipocyte and macrophage function in normal and pathophysiological conditions (inflammation, type-2 diabetes, obesity, atherosclerosis) [1]. Its expression in mesenchymal stem cells also associated this receptor with bone, skin, and muscle differentiation [2]. This 50-kDa protein consists of (from N- to C-terminal) the following: a transactivation function-1 (AF1) harbouring an MAPK-phosphorylation motif PXSP, a zinc-finger-type DNA-binding domain (DBD), a hinge region, the ligand-binding domain (LBD), and a flexible AF2 helix. Ligand-binding triggers the formation of the “charge clamp” between the AF2 and the core LBD, an event that enables the release of NR corepressors (NCoRs), heterodimerization with RXR, DNA-binding, NR coactivator (NCoA) recruitment, and transactivation of promoters [29] (Figure 1(a)). The LBD/AF2 interface also constitutes an important docking interface with unusual coregulators such as kinases and cell-cycle regulators (reviewed in [32]).

PPAR*γ* positively regulates the expression of a vast spectrum of target genes involved in immunity and inflammation, differentiation, proliferation, apoptosis, cell survival, and metabolism [10]. However, PPAR*γ* can also repress transcription by negatively interacting with several proinflammatory [9] and promitotic transcription factors [33] such as ETS, STAT, AP1, and NF*κ*B (Figure 1(c)). Thereby, this factor promotes terminal differentiation of various normal and transformed cells of epithelial and mesenchymal origin. PPAR*γ*(−/+) knockout mice exhibit enhanced susceptibility to chemically induced tumorigenesis [34, 35], and this enhanced susceptibility is observed also upon breeding with other strains deficient in tumor suppressors (such as APC) [36]. In patients, PPAR*γ* protein is expressed (in varying levels) in leukemias, lipo- and osteosarcomas and in many carcinomas. Gene polymorphisms within the human population result in several “loss-of-function” PPAR*γ* variants that are associated with metabolic diseases (insulin resistance, lipodystrophy) [37] and cancers (e.g., colon, stomach) [4, 5, 38, 39]. These data initially corroborated PPAR*γ* as a protective transcription factor.

In line with the latter findings, ERK- (and other MAPK-) mediated phosphorylation of PPAR*γ* reduces its genomic activity. A panel of extracellular/environmental promitotic, stress and inflammatory stimuli (growth factors, hormones, cytokines, lipid mediators/eicosanoids, UV-radiation, anisomycin, acetaldehyde, etc.) trigger the activation of the MAPK-family members: ERK, JNK, and p38 (Figure 1(a)). These MAPKs phosphorylate (in humans) Ser 84 in the PPAR*γ*1 and Ser 114 in PPAR*γ*2 isoform, which correspond to Ser 82/112 in mouse and are both located in the AF1 region of the molecules. This phosphorylation results in suppression of the PPAR*γ*’s ability to transactivate target gene promoters and thereby its physiological functions (reviewed by [40, 41]). In addition, phosphorylated PPAR*γ* is assumed to be more prone to other posttranslational modifications (sumoylation, ubiquitination) and subsequent degradation by the proteasome, an event that promotes its further downregulation upon MAPK-activation [42, 43]. But these effects are not fully characterized yet. In any event, the inhibition of PPAR*γ* activity by MAPK phosphorylation is in accordance with the anti-inflammatory and prodifferentiation action of PPAR*γ* and has been verified for normal (fibroblasts, adipocytes, macrophages, hepatic stellate cells) as well as cancer cell lines, various stimulating agents (as reviewed in [31, 44]) and also in vivo [45, 46]. An additional level of crosstalk is constituted by the fact that PPAR*γ* cofactors, such as steroid receptor coactivator (SRC) family members (e.g., AIB/SRC3 in breast cancer), are phosphorylated by MAPKs and thereby are altered in their ability to coactivate transcription [47] (Figure 1(c)).

The effect of PPAR*γ* phosphorylation by MAPKs was also supported by several in vivo studies. For example, a “knock in” of an unphosphorylable allele S112A in mice preserved their insulin sensitivity in absence of lipogenesis (weight gain) in a setting of diet-induced obesity [45]. In addition, a recent study revealed “downstream of tyrosine kinases-1” (Dok1) as an adapter protein in the insulin-signaling pathway that inhibits S112 phosphorylation of PPAR*γ*2 in vivo [46]. Dok1 knockout mice on high fat remain lean and insulin-sensitive, and Dok1 knockout mouse embryonal fibroblasts (MEFs) show defective adipogenic differentiation, increased ERK activation and phosphorylation of PPAR*γ*2 on S112. Mutation of S112 of PPAR*γ*2 blocked the lean phenotype in Dok1 knockout mice, indicating that Dok1 promotes adipocyte growth and differentiation by counteracting the inhibitory effect of ERK on PPAR*γ*. Another current intriguing example is the identification of parvin*β*, a focal adhesion protein (lost in breast cancer patients), that increases the expression, S84 phosphorylation, and activity of PPAR*γ*1 through cyclin-dependent kinase 9 (CDK) and suppressed breast cancer growth in vivo [48]. These data indicate that MAPK-mediated S84/S114 phosphorylation alters the activity of PPAR*γ*1/2 in vitro and in vivo.

In sum, these studies initially corroborated the role of PPAR*γ* as a tumor suppressor [2, 14], which may be shut down by MAPK-phosphorylation [44]. However, more recent evidence was collected, that PPAR*γ* is a context-specific tumor modulator, whose effector profile is complemented and modified by PPAR*γ*-independent effects of its ligands (e.g., TZDs and eicosanoids) and by reciprocal regulation of PPAR*γ* through members of the ERK cascade as follows [31, 40].

### 2.2. PPAR*γ* ligands influence cellular processes via a nongenomic activation of the ERK cascade

A second mechanism of crosstalk between PPAR*γ* and the ERK cascade comprises the direct activation of ERKs by PPAR*γ* ligands. In the past, ample data was collected on the effects of chemically distinct classes of PPAR*γ* ligands on cells. Different ligands induce either cell growth and proliferation or growth arrest and apoptosis in various human and mouse cancer cell lines and xenografts (as extensively reviewed in [14, 31]), and also modulate angiogenesis in vitro and in vivo [49]. These effects are dose-, time-, and cell type-dependent, and manifest either in a PPAR*γ* receptor-dependent (“genomic”) or non-PPAR*γ* receptor-mediated (“nongenomic”) manner or in a combination of both. The mechanisms that underlie these context-dependent responses are largely unknown. One concept is based on the claim that nongenomic PPAR*γ* ligand effects manifest at higher micromolar concentrations (>10 *μ*M) well above the low EC50’s necessary for classical genomic actions on PPAR*γ*/RXR heterodimers at characterized PPREs in target genes (e.g., 80 nM for rosiglitazone) [50, 51]. This assumption translated into the idea that, low doses of PPAR*γ* ligands, for example, that correspond to the pharmacological doses prescribed for diabetic patients, exert overtly beneficial efficacy, while supra-pharmacological high doses evoke adverse effects. For example, troglitazone was retracted from the market due to hepatotoxicity, which was not a TZD-class effect but due to a drug-specific (possibly “nongenomic”) adverse action [2]. However, the literature provides examples for both pro- *and* antitumor actions of PPAR*γ* ligands at similar dose ranges in similar cellular systems. Thus, an underlying principle for the separation of genomic from nongenomic PPAR*γ* ligand effects is currently not available.

The PPAR*γ* ligand effects are likely to be mediated either (i) through so far unknown plasma membrane-bound receptors (Figure 1(d)) or (ii) through cytoplasmatic localized PPAR*γ* protein (Figure 1(b)). Novel G-protein coupled receptors, such as GPR30 for estradiol [52], TGR5 for bile acids [53], and GPR40 for free fatty acids [54], were identified to function as alternative signal transducers for NR-ligands. GPR40, a candidate PPAR ligand receptor, is highly expressed in the pancreas but also in monocytes and in the lower GI tract (e.g., ileum, colon) [55, 56]. Oleate, a natural PPAR ligand, increases proliferation of MCF7 human breast adenocarcinoma through binding and signaling via endogenous GPR40 [57]. TZDs were postulated as bona fide ligands for ectopic GPR40 in CHO cells and to signal via G*α*i/q proteins, cAMP, calcium, and ERK activation [58]. However, in vivo proof is lacking. In addition to GPCRs, also plasma membrane-bound classical NRs interact with specific adapter or scaffold proteins in the cytoplasm and trigger the initiation of proproliferative and survival signaling [59]. For example, the estrogen receptor docks to modulator of non-genomic action of estrogen receptor (MNAR) that recruits Src and leads to activation of the p85 subunit of PI3K [60] and the ERK cascade [61]. If this situation is also relevant for PPAR*γ* molecules remains to be shown. Many TZD effects actually target cytoplasmic proteins such as at mitochondria, the proteasome, or the translational machinery. Thus, it is possible that cytoplasmic PPAR*γ* molecules are also involved in the transduction of “nongenomic” TZDs signals.

Downstream of the initial ligand triggering event, nongenomic responses to PPAR*γ* ligands include transient alterations in mitochondrial functions and activation of stress (production of reactive oxygen species (ROS)) as well as kinase signaling pathways promoting proliferation and survival such as PI3K-PKB/AKT, ERK, p38, and JNK [50, 51]. Rapid signaling initiated by ligands can be mediated by membrane proximal events such as cleavage of transmembrane proteinases (ADAMs), activation of GPCRs, EGFR transactivation, calcium influx, and activation of protein tyrosine kinases (Pyk2, Src). Further downstream effects include PPAR*γ*-independent induction of “early response genes” such as c-Fos and Egr-1. In this context, it was shown that PPAR*γ* ligands enhance proliferation, survival and drug resistance in cancer cells, for example, by induction of the prosurvival and promitotic hormone gastrin [62]. We showed that TZDs enhance drug resistance in human colon adenocarcinoma HT29 cells in a PPRE-independent but EGFR-dependent manner, involving Src/MAPK-signaling [63]. In colon carcinoma cells, TZDs induce matrix metalloproteinase 2 (MMP2) and membrane type 1-MMP (MT1-MMP) activation and concomitantly increase tumor cell invasion through generation of ROS and activation of the ERK cascade [64]. On the other hand, ERK cascade activation by TZDs may also translate into growth inhibition and/or apoptosis [65–69]. It is currently unknown which mechanism governs the decision for pro-versus antiproliferative responses upon TZD application.

In addition to TZD drugs, also the physiological eicosanoid-type ligands for PPAR*γ* exert tumor-modulating effects through their ability to trigger ERK cascade activation [70]. Eicosanoids are generated by cytoplasmic phospholipase A2 and cyclooxygenases (COX1/2). Some of these arachidonic acid metabolites act as endogenous PPAR*γ* ligands ((e.g., 15-deoxy-Δ(12,14)-PGJ2) [71]), while others, like the prostaglandins of the E and D series, activate the ERK cascade through prostanoid GPCRs at the cell membrane [72]. 15-deoxy-Δ(12,14)-PGJ2 directly inhibits inhibitor-*κ*B kinase (IKK) in an intracellular fashion and exerts various effects on inflammation, cell growth, and apoptosis independent of a prostanoid GPCR [71]. For example, in human breast MCF7 adenocarcinoma cells, 15-deoxy-Δ(12,14)-PGJ2 upregulates VEGF synthesis through induction of heme oxygenase-1, an enzyme that stimulates proliferation and angiogenesis, and triggers ERK phosphorylation in an PPAR*γ*-independent fashion [73]. In sum, these data point out to the important role for protumor effects of PPAR*γ* ligands of the TZD- and eicosanoid-class in the activation of ERK cascade-related proliferation and survival pathways, which stand in sharp contrast to the otherwise reported tumor suppressive effects of the latter in similar cellular systems [65–67].

In vivo preclinical and clinical data of TZDs support the concept of an overlapping profile of PPAR*γ* receptor-dependent and independent ligand signaling. In contrast to the lessons from PPAR*γ*(+/−) knockout mice [34, 35] and the antineoplastic action of PPAR*γ* receptor activation in vitro [33], ample in vivo data asserted that many potent and selective PPAR*γ* ligands actually promote tumorigenesis. Thus, PPAR*γ* ligands induce tumor growth in rodent xenograft models [14] and enhance in vivo angiogenesis [49]. In addition, TZDs act as procancerogenic agents in wild-type and APC-deficient mouse models of colon carcinogenesis [74–77]. Importantly, clinical studies in humans failed to show a clear benefit of TZD monotherapy in cancer patients [14, 78, 79]. PPAR*γ* ligands are procarcinogenic in human bladder, as evaluated by the PROactive study [12], and in the rodent bladder [80, 81]. As a reaction towards the safety-toxicological data collected in preclinical studies and clinical trials regarding TZD use, the US Food and Drug Administration (FDA) (http://www.fda.gov/cder/present/DIA2004/15) issued a warning of tumor-related adverse effects of novel potent PPAR*γ* ligands that are currently in clinical trials as novel antidiabetics or obesity cures (reviewed in [82]) [83, 84]. The FDA classified all PPAR*γ* ligands as multispecies and multiorgan carcinogens requiring strict dose finding for therapeutical use in humans. However, the full molecular mechanism of this interplay between tumor promoting versus suppressing action of PPAR*γ* ligands is so far unknown.

### 2.3. Towards solving the tumor initiation/suppression paradox of PPAR*γ*: interaction of PPAR*γ* with the ERK cascade in cancer

Unlike the impression that is left by many articles to date, PPAR*γ* protein does not always act as a tumor suppressor, and the PPAR*γ* ligands are not always procancerogenic independently of the receptor. Notably, the PPAR*γ* itself seems to be important for exacerbating mammary gland tumor formation in bitransgenic mice expressing a constitutive active PPAR*γ* form independently of application of an exogenous ligand [85]. An interesting in vitro study corroborated the functional cooperation of the PPAR*γ* receptor and the ERK cascade in the promotion of epithelial-mesenchymal transition (EMT) in the mouse small intestine and rat intestinal epithelial cells, which was dependent on an intact DNA-binding activity of the PPAR*γ* receptor protein [86]. In this system, PPAR*γ* induced ERK1/2 phosphorylation by activating PI3K, Cdc42, and p21-activated kinase (PAK), which in turn phosphorylated S298 of MEK1 that supports its activity [23]. Ectopic expression of dominant negative MEK1 blocked EMT induced by PPAR*γ*, while constitutively active MEK1 overexpression promoted a mesenchymal morphology. However, as evident in the latter intriguing example, the exact molecular mechanisms and physiological relevance of the cooperative interactions between posttranslational regulation of NRs by kinases and rapid nongenomic kinase activation by NR-ligands are so far unknown.

Ample data supports the notion that mutual physical/allosterical associations between kinases and NRs exist that translate into reciprocal regulation of their activities [87, 88]. For example, 3-phosphoinositide-dependent protein kinase-1 (PDK1), that is the upstream activator of AKT/PKB, binds to and activates PPAR*γ* during adipogenic differentiation [89]. Complexes of cyclins and CDKs are cofactors for and phosphorylate PPAR*γ* in adipocytes [90, 91]. PPAR*γ* also interacts with and is activated by ERK5 [92, 93] in order to inhibit (in conjunction with WNT signaling factors) the proliferation of lung cancer (NSCLC) cells and inflammation in endothelial cells upon flow (shear stress), indicative of a protective function of ERK5-PPAR*γ* cooperation. These unusual NR cofactors [32], that also include retinoblastoma protein and transcriptional elongation factors, directly interact with regulatory domains in NRs and considerably add to the pleiotropic effector profile of a given NR. Several interaction partners for PPAR*γ* protein have been identified including prominent oncogenic modulators such as *β*catenin [94, 95] and MEK1 [30]. Therefore, it is likely that PPAR*γ* interacts with or cooperates with several signaling pathways and particularly the ERK cascade in order to induce or prevent oncogenic transformation dependent on the cell type and environment.

#### 2.3.1. Spatial regulation of PPAR*γ* activity: MEKs export PPAR*γ* to the cytoplasm

Next to Ser84/114 phosphorylation and the nongenomic ERK activation by PPAR*γ* ligands, the direct interaction of PPAR*γ* with the ERK cascade component MEK1 constitutes a third mechanism of crosstalk between PPAR*γ* and the ERK cascade. Subcellular compartmentalization is a major mechanism in regulating cellular signaling. Interestingly, PPAR*γ* itself can regulate the membrane translocation of other proteins such as NF*κ*B in gut intestinal epithelial cells [96] and PKC in macrophages [97]. Several reports have demonstrated a signal-mediated translocation of PPAR*γ* between the nucleus and the cytoplasm in vitro (as reviewed in [98]). In addition, it was shown that PPAR*γ* is expressed predominantly in the nucleus of nonneoplastic tissues, whereas it is present in both the nucleus and the cytoplasm of tumorous tissues in squamous cell carcinoma (SCC) of the lung, indicative of a correlation of malignancy with differential PPAR*γ* compartmentalization [99]. Moreover, a dominant negative PPAR*γ* splice variant was described in lung SCC patients, an event that leads to the loss of apoptosis sensitivity in response to oxidative stress and cisplatin [99]. Differential compartmentalization of PPAR*γ* was also described in gastric cancer patients [100]. The ratio of cytoplasmic/nuclear PPAR*γ* expression decreased in the progression of intestinal metaplasia to undifferentiated cancers [100]. In salivary duct carcinoma, an aggressive tumor type, PPAR*γ* is highly expressed (80%) and topographically located in the cytoplasm [101], indicative of an inactivation of its genomic activities in the nucleus. Cytoplasmic PPAR*γ* was also detected in the cytoplasm (58%) of infiltrating breast carcinoma samples and was proposed as an independent prognostic factor for patients with ductal carcinoma [102]. However, the function of this subcellular distribution of PPAR*γ* molecules are yet unknown.

The mechanism that may induce the changes in localization of PPAR*γ* upon stimulation, or upon neoplastic transformation was only recently elucidated by us [30]. We showed that PPAR*γ* is exported from the nucleus to the cytoplasm by MEK1/2. This is induced by a reversible interaction of PPAR*γ* with MEK1 through association of the AF2 of the first with the N-terminal docking domain of MEK1. This export to the cytoplasm (Figure 1(b)) leads to reduction in its genomic function in the nucleus [30]. We also elucidated the molecular mechanisms of the export and the physiological implications, but the question remained is whether cytoplasmatically located PPAR*γ* is subjected to degradation or shunted to alternative signaling compartments such as lipid droplets, ER/Golgi, cytoskeleton, or the plasma membrane. To this regard, we tend to speculate that alternative locations of PPAR*γ* in the cell may determine the balance between tumor-suppressive and tumor-promoting functions.

#### 2.3.2. Tumor-suppressive functions of PPAR*γ* related to ERKs and MEKs interaction

Due to the coexpression of the ubiquitous proteins PPAR*γ* and MEK1/2 in different organs of the body, it was interesting to identify their coregulation in various physiological and pathological processes, as described below.

**Differentiation**

Due to the lethality of MEK1 knockout mice [103] and absence of phenotypes in MEK2 knockout mice [104], the major focus of interest was directed towards the role of MEK1 overexpression in vivo. Constitutively active MEK1 (S218E/S222E) has been conditionally overexpressed (among other tissues) in the skin and bone of mice [105]. All transgenic mice exhibited increased cell numbers (hyperplasia) and cell size and a defect in terminal differentiation. Interestingly, both in skin and in bone of mice, PPAR*γ* was shown to be an important player promoting differentiation [2]. In addition, the constitutively active MEK1 overexpressing mice show dwarfism and reduced bone size due to defective ossification and impaired chondrocyte differentiation. In other systems, it was shown that osteoclast-specific PPAR*γ* knockout mice are characterized by increased bone mass due to impaired osteoclast differentiation [106], suggesting antagonistic effects of PPAR*γ* and MEK1 on different bone cell types: with PPAR*γ* promoting osteoclast differentiation, and MEK1 inhibiting chondrocyte differentiation.

Skin-restricted MEK1 transgenic mice exhibit hyperproliferation, hyperkeratosis and of age papillomas at sites of wounding [105, 107]. Vice versa, epidermis-specific knockout of MEK1/2 in mice [108] resulted in hypoproliferation, apoptosis, skin barrier defects, and death, indicative of a positive role of MEK1 in skin proliferation and tissue homeostasis. PPAR*γ* knockout mice are characterized by an increased sensitivity to experimentally-induced skin tumors [35], emphasizing the tumor suppressor and differentiation promoting activity of PPAR*γ* in the skin. These “mirror-images” phenotypes in the organs where MEK1/2 and PPAR*γ* are normally coexpressed may give some indication for the antagonistic regulation of the two proteins, MEK promoting proliferation and dedifferentiation, PPAR*γ* promoting terminal differentiation. In line with this idea, it was shown that the kinase activity of MEK1 was actually dispensable for the hyperproliferative and integrin-inducing effects of the MEK1 in mouse skin [109]. Instead, a kinase-dead mutant of MEK1 elicited the same phenotype, indicative of an involvement of other MEK1-functions such as scaffolding inhibition of differentiation-promoting cellular factors.

In adipogenic differentiation systems originating from (mesenchymal) stem cells, synergistic cooperations between the MEK-ERK cascade and PPAR*γ* have been described. In fibroblasts, differentiating towards the adipogenic lineage, a positive cooperation between PPAR*γ* and MEK1 exists that facilitates the adipogenic program by MEK1-dependent induction of the C/EBP*α* gene [110]. In bone marrow-derived mesenchymal stem cells isolated from normal and streptozotocin (STZ)-induced diabetic FVB/N mice, high glucose enhanced adipogenesis, lipid accumulation, and PPAR*γ* expression via PI3K/AKT and ERK cascade signaling, events that were all inhibited by the MEK-inhibitor PD98059 [111]. In differentiated C2C12 myocytes, the free fatty acid palmitate reduces the mRNA levels of PPAR*γ*-coactivator-1*α* (PGC1*α*) and activated MEK, while the MEK inhibitors PD98059 and U0126 prevented such downregulation of PGC1*α*, indicative of a MEK-mediated inhibition of an important NR coactivator protein for PPAR*γ* in muscle cells [112]. These findings corroborated that the MEK-ERK cascade and PPAR*γ* signaling pathways can syn- or antagonistically cooperate to control the balance of proliferation and differentiation in an organ/cell type-specific manner.

**Cell cycle**

The ERK cascade participates in the regulation of cell cycle at (i) G0/G1 and G1/S transitions in response to mitogenic stimulation (as reviewed in [24]) and (ii) in the process of Golgi fragmentation [113–115] during mitosis. This is mediated in part by the nuclear translocation of ERK upon cellular stimulation that promotes expression of “immediate early” genes such as members of the AP1 family that activate the promoters of the G1 cyclins D and E. However, the subcellular compartmentalization of ERK signaling by scaffold proteins (KSR, MP1/p14, Sef) (reviewed in [22]) indicates a novel mode of spatial separation of substrate specifities and signal translation. For example, MP1 via the adapter protein p14 tethers MEK1 to endosomes [116] and focal adhesions [117]. Sef translocates MEK1 to the Golgi apparatus, prevents nuclear translocation of ERKs, and, thereby, favours phosphorylation of cytoplasmic ERK substrates instead of nuclear ones [118]. The latter subcellular localization-determining systems may thus be as well exploited by the PPAR*γ*-MEK1 nuclear export shuttle to regulate the cell cycle.

The PPAR*γ* receptor has been involved in the inhibition of the G0/G1-transition by up-regulation of genes coding for the CDK-inhibitors p18(INK4C) [119] and p21(WAF1/CIP1) [120, 121], and in the inhibition of G1/S transition through upregulation of the p27(KIP1) gene [122, 123]. Upregulation of other genes implicated in cell cycle control such as PTEN or members of the BCL-gene family contributes to the growth-arresting and/or apoptosis-inducing action of PPAR*γ* ligands [15]. The cell cycle modulatory actions of PPAR*γ* are usually not mediated through classical PPRE binding at the DNA but rather through PPRE-independent “off-DNA” crosstalk to other transcription factors [15] and through nongenomic effects in the cytoplasm, such as inhibition of translation initiation [124, 125] and modulation of the proteasomal machinery [126–128]. The latter processes may be mediated by ligand-activated cytoplasmic PPAR*γ* molecules or cytoplasmic alternative signal-transducers for PPAR*γ* ligands. We therefore hypothesize that, by nuclear export and cytoplasmic retention of PPAR*γ*-MEK1 complexes to other MEK1-scaffolding locations (e.g., at the Golgi, endosomes, focal adhesions), the genomic PPAR*γ* functions may as well be redirected in favour of cytoplasmic signaling events. In sum, the cell cycle modulating effects of PPAR*γ* protein and its ligands may be caused by its differential subcellular compartmentalization by MEK1.

#### 2.3.3. Tumor-promoting functions of PPAR*γ*, related to crosstalk with the ERK cascade

**Metastasis**

In contrast to the initial assumption of PPAR*γ* mainly acting as a tumor suppressor whose activity and/or expression is lost in cancers, PPAR*γ* expression and activity can also be a negative predictor of cancer aggressiveness; and positive cooperation between PPAR*γ* and components of the ERK cascade in malignant phenotypes takes place. For example, strong nuclear PPAR*γ* expression was detected in thyroid carcinomas compared to normal tissue, and patient samples of thyroid carcinoma-associated lymph node metastasis also showed a higher percentage of PPAR*γ*-positive staining than other case categories [129]. PPAR*γ* expression was also elevated in human prostate cancer compared to normal prostate [130]. In patients with invasive breast carcinoma, cytoplasmic MT1-MMP and MMP9 expression positively correlated with PPAR*γ* levels [131]. These data corroborated a positive relationship between PPAR*γ* expression and malignancy state in certain tumor entities, a fact that was shown to be therapeutically exploitable by the use of PPAR*γ* antagonists or siRNA. This was described in primary esophageal tumor specimen and in esophageal cancer cell lines [132], in human primary squamous cell carcinoma (SCC) and lymph node metastases [133] and in hepatocellular carcinoma (HCC) samples [134], where PPAR*γ* expression is elevated compared to matched normal tissue. In all three cell systems, PPAR*γ* antagonists (T0070907,GW9662) and RNAi-mediated knock-down of PPAR*γ* levels reduced the invasiveness and adherence of cells to the extracellular matrix, triggered anoikis, or inhibited proliferation by decreasing the phosphorylation status of focal adhesion kinase (FAK), MEK, and ERK. Therefore, in tumors where elevated PPAR*γ* and activated ERK and MEK levels contribute to the malignant phenotype, inhibition of PPAR*γ* may be beneficial as a therapeutic strategy (see also Section 3).

**Angiogenesis**

The overall vascular protective and antiatherogenic effects of PPAR*γ* ligands provide essential add-ons for the clinical application as insulin sensitizers (reviewed in [13]). However, the proangiogenic effects of PPAR*γ* ligands via modulation of the VEGF/VEGF-receptor system (that signals via the ERK cascade) have gained recognition (reviewed by [49]), which may be beneficial for therapy of vascular diseases (e.g., infarction) [135, 136] but detrimental in cancer tissue. For example, in rat myofibroblasts, rosiglitazone and 15-deoxy-Δ(12,14)-PGJ2 induce expression of VEGF and its receptors (Flt1 and KDR, that signal via the ERK cascade), and augment tubule formation on a matrigel, indicative of a promoting function of PPAR*γ* and ERKs in angiogenesis [137]. In osteoblast-like MC3T3E1 cells, pioglitazone and ciglitazone augmented FGF2-induced VEGF release in a PPAR*γ*-dependent manner and enhanced the phosphorylation of JNK [138]. In human RT4 bladder cancer cells, VEGF mRNA and protein are upregulated by PPAR*γ* via activation of the VEGF promoter. Interestingly, the MEK inhibitor PD98059 reduced PPAR*γ* ligand-induced expression of VEGF [139], indicative of a positive cooperation of PPAR*γ*-ERK pathways in angiogenesis. These positive effects on angiogenesis were examined also in two clinical studies with rosiglitazone [140] and pioglitazone [136], in which it was demonstrated that chronic addition of the TZDs increased endothelial cell precursor counts and migration in diabetic patients, raising concern on the proangiogenic potential of TZDs.

Taken together, the data which revealed an antagonistic cooperation of PPAR*γ* and ERK signaling in several cell or tissue-specific differentiation systems (skin, bone, muscle, fat) is now challenged by the findings of positive cooperation of the same components in tumor progression (metastasis, angiogenesis). Thus, the role of PPAR*γ* as a MEK/ERK-regulated tumor suppressor seems to be of importance in normal tissue or in prevention of tumor initiation, while in advanced stages of certain tumors a synergistic cooperation between PPAR*γ* and the ERK cascade may contribute to the malignancy of the disease. Future studies have to clarify whether PPAR*γ* agonists, PPAR*γ* antagonists, or PPAR*γ* modulators/partial agonists (SPPARMs) with a selective effector profile [141] may be of interest for the therapy of certain tumor entities.

## 3. CLINICAL USE OF PPAR*γ* INTERACTION WITH THE ERK CASCADE AS A DRUG TARGET

**Figure 2:**
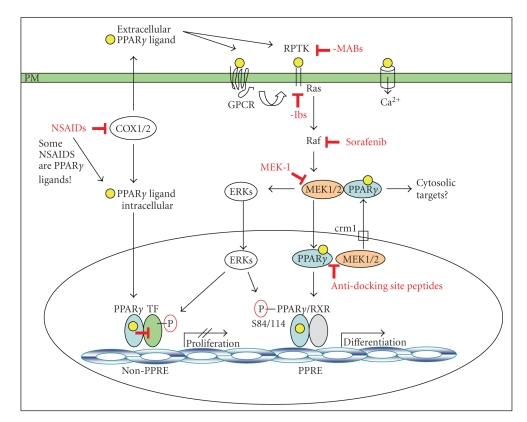
*Model of the combination therapy using PPAR*γ* ligand and ERK cascade inhibitors*. The simultaneous inhibition of EGF receptor-initiated ERK cascade activation by specific kinase inhibitors (-ibs) or antibodies (-MABs) and supply of PPAR*γ* ligands (in tumors that have a need for restored PPAR*γ* activity) will avoid: (a) ERK-mediated downregulation of PPAR*γ* through Ser84/114 phosphorylation, (b) MEK1-driven nuclear export and cytoplasmic retention of PPAR*γ*, (c) activation of prosurvival and proproliferative ERK cascade signaling by exogenous PPAR*γ* ligands (e.g., by TZD drugs) or endogenous eicosanoid type of PPAR*γ* ligands (e.g., generated by COX1/2), but is expected to (d) restore the differentiation-inducing and proapoptotic functions of PPAR*γ* and its ligands, and (e) promote the transrepressive activity of PPAR*γ* on other promitotic and proinflammatory transcription factors (e.g., AP1, ETS, STAT, NF*κ*B). *Legend*: Yellow circles = PPAR*γ*-ligand; TF = transcription factors; ROS = reactive oxygen species; GPCR = G protein coupled receptor; RPTK = receptor protein tyrosine kinase; crm1 = exportin1; NSAID = nonsteroidal anti-inflammatory drug; COX = cyclooxygenase; -Ibs = LMW tyrosine kinase inhibitors; MABs = monoclonal tyrosine kinase antibodies.

Reactivation (“differentiation”) therapy targeting functional PPAR*γ* protein in cancer cells/tissues by exogenous application of TZD-class PPAR*γ* ligands was lately expected to represent a novel approach to fight cancer [142]. However, differentiation-inducing monotherapy with TZDs did not show the expected clinical benefit [11]. Instead, evidence accumulated that alternative (“nongenomic”) PPAR*γ* signaling pathways, crosstalk with the ERK cascade and elevated PPAR*γ* expression levels in certain tumor types (where PPAR*γ* is postulated to act as a prosurvival factor, e.g., in hepatocellular carcinoma, squamous cell carcinoma), are the cause for the observed tumor promoting effects of PPAR*γ* ligands, and may explain the absence of clear therapeutical benefit of TZDs in cancer patients [78, 79, 143]. Therefore combination therapy of PPAR*γ* ligands with kinase inhibitors may represent a novel strategy to circumvent the crosstalk of PPAR*γ* and ERK cascade signaling and limit PPAR*γ* protein activation to its classical differentiation-inducing feature (Figure 2). This dual approach is expected to avoid (a) ERK cascade-mediated downregulation of PPAR*γ*, (b) MEK-driven nuclear export and cytoplasmic retention of PPAR*γ* and (c) nongenomic amplification loops of PPAR*γ* ligands towards the ERK cascade, but to promote (d) the growth-arresting and proapoptotic genomic functions of PPAR*γ* and its ligands, and (e) the negative crosstalk of PPAR*γ* with promitotic and proinflammatory transcription factors in the nucleus. This concept may not be suitable for tumor types with elevated “malignant” PPAR*γ* expression/activities. However, due to the lack of clinically approved PPAR*γ* antagonists, no statement can be currently made on the potential therapeutical benefit of PPAR*γ* and kinase coinhibition.

### 3.1. In vitro studies

The combination of PPAR*γ* ligands and inhibitors against receptor tyrosine kinases of the EGFR-family or cytoplasmic tyrosine kinases (e.g., Abl) revealed some promising results in leukemia and carcinoma cells. Gefitinib, an inhibitor of the EGFR/Her1 kinase, exhibits antitumor activity in only a fraction of 10–20% of patients with nonsmall cell lung cancer (NSCLC) [144]. The mechanisms underlying this resistance to gefitinib are not known. However, application of rosiglitazone reduced the growth of the NSCLC A549 cells and potentiated the antiproliferative effects of gefitinib and increased PPAR*γ* and PTEN expression in these cells, indicative of a potential benefit of this drug combination also in cancer patients. MCF7 breast cancer cells stably transfected with ErbB2/Her2 displayed reduced differentiation and enhanced resistance to TZD-driven inhibition of anchorage-independent growth [145]. Herceptin, a monoclonal antibody against Her2 kinase, sensitized cells for the differentiation-promoting and growth-inhibitory effects of troglitazone. This concept also held true for chronic myeloid leukemia (CML) cell lines, where TZD18 (a dual PPAR*α*/*γ* ligand) enhanced CDK-inhibitor p27(KIP1) expression and inhibited cyclin E, cyclin D2 and CDK2 [122]. TZD18 synergistically enhanced the antiproliferative and proapoptotic effect of imatinib, a clinically used kinase inhibitor of the Bcr-Abl fusion protein. Collectively, this work demonstrated that the targeting of receptor tyrosine kinase signaling with LMW inhibitors or monoclonal antibodies can improve the sensitivity of cancer cells to PPAR*γ* ligand-mediated growth inhibition.

### 3.2. In vivo rodent and clinical studies

The clinical outcome of selective MEK inhibitors in patients studies was disappointing (CI-1040, PD0325901, AZD-6244) (reviewed in [146, 147]). On the other hand, a Raf inhibitor, sorafenib, was recently approved for clinical use; and novel selective Raf inhibitors are under development [148]. So far no clinical studies were performed using MEK or Raf inhibitors in combination with PPAR*γ* ligands. However, successful treatment data in mouse models or patients are available for combinations of PPAR*γ* ligands and three other types of inhibitory drugs: classical chemotherapeutics, COX-inhibitors (NSAIDs), and established tyrosine kinase inhibitors (imatinib, gefitinib, herceptin).

NSAID/COX-inhibitors have been shown to reduce the risk for colon carcinoma formation, however at the expense of gastric ulcer and cardiovascular complications [19]. Several NSAIDs are also low-affinity PPAR*γ* ligands, a fact that led to the speculation that a part of the clinical profile of these compounds is related to low-level activation of PPAR*γ* [19]. Therefore, clinical trials with combination therapies were initiated to exploit PPAR*γ* activation and simultaneous blockage of the promitotic and proinflammatory COX1/2-mediated eicosanoid production, which contributes to nongenomic signaling in cancer tissues (Figure 2). Pilot clinical studies with an angiostatic triple combination of pioglitazone, rofecoxib (a selective COX2 inhibitor), and trofosfamide showed benefit in patients with angiosarcoma and hemangioendothelioma [151, 152] and advanced sarcoma [153]. A phase-II trial with the same triple combination in patients with metastatic melanoma or soft-tissue sarcoma evinced disease stabilization [152], indicative of a beneficial effect of COX2 inhibition (whose eicosanoid metabolites activate the ERK cascade) and simultaneous PPAR*γ* activation in sensitization of tumor cells to differentiation and/or apoptosis. A recently published outcome of a phase-II trial in high-grade glioma patients (glioblastoma or anaplastic glioma) under pioglitazone and rofecoxib combined with chemotherapy (capecitabine or temozolomide) also stated some disease stabilization [157]. However, due to the severe side effects of selective COX2-inhibitors this therapeutic regimen may raise concerns.

Preclinical studies in rodents provided evidence for a therapeutic potential of combination therapy with other inhibitory agents. In mice xenografted with NSCLC A549 cells, the PI3K inhibitor PX-866 potentiated the antitumor activity of gefitinib [149]. The glucose intolerance related to PX-866 in mice was reversed by insulin and pioglitazone. PX-866 in combination with insulin sensitizers may thus be useful in facilitating the response to EGFR inhibition. The antitumoral action of rosiglitazone on experimentally induced mammary tumors induced by N-nitroso-N-methylurea (NMU) in Sprague-Dawley rats was potentiated by the selective estrogen-receptor modulator (SERM) tamoxifen with respect to the extent of tumor cell apoptosis and necrosis [150]. The PPAR*γ* ligand RS5444 in combination with paclitaxel had additive antiproliferative effect in vitro and minimized tumor growth in nude mice xenografts of anaplastic thyroid carcinoma (ATC) cells [120]. These preclinical studies underline that the combination of PPAR*γ* ligands and established anticancer drugs may be of clinical benefit also in cancer patients.

Interestingly, two studies provided already first-line evidence for the potential of an in vivo reactivation of PPAR*γ* protein function by simultaneous inhibition of the COX pathway-mediated activation of the ERK cascade: LY293111, an oral PPAR*γ* ligand, leukotriene B4 receptor antagonist and 5-lipoxygenase inhibitor, was validated for its antineoplastic efficacy in combination with chemotherapy (irinotecan, gemcitabine) in preclinical models [154] and evoked disease stabilization in patients with advanced solid tumors [155, 156]. The NSAID R-etodolac inhibits growth of prostate cancer (CWRSA6, LuCaP35) xenografts in mice by downregulation cyclin D1. However, the combination of R-etodolac with herceptin elicited an additive antitumor effect, reduced ERK phosphorylation and stabilized PPAR*γ* protein levels [158]. These therapeutic regimens inhibited the eicosanoid-mediated activation of the ERK cascade, and in conjunction with PPAR*γ* activation, may provide a basis for differentiation-inducing therapy in combination with classical chemotherapeutics or biologicals.

So far no clinical evidence was published on the combined use of ERK cascade inhibition and PPAR*γ* activation (in tumors with low PPAR*γ* expression/activity) or PPAR*γ* inhibition (in tumors with high PPAR*γ* expression/activity). In the future, the combination of PPAR*γ* ligands with kinase inhibition selectively targeted by MABs against the EGFR tyrosine receptor kinase family or LMW selective inhibitors of the downstream ERK cascade, such as Raf and MEK, may constitute a possible new approach to treat cancer.

## 4. CONCLUSION AND PERSPECTIVES

In conclusion, PPAR*γ* emerges as a tumor-type and tumor-stage-specific modulator that is regulated by at least three mechanisms through the ERK cascade. Downregulation is carried out through (1) MAPK-mediated Ser84/114 phosphorylation, (2) ERK cascade activation through PPAR*γ* ligands, and (3) cooperation of PPAR*γ* with tumor modulating proteins (such as MEK1). The overlay of these 3 mechanisms of crosstalk is likely to determine the physiological outcome of PPAR*γ* effector functions. Consequently, interference with these interactions by LMW inhibitors, antibodies, or peptidomimetic drugs against protein docking interfaces may constitute a novel approach to redirect PPAR*γ* effector functions from a protumorigenic towards an antitumorigenic profile. Simultaneous inhibition of ERK cascade-mediated signaling is expected to prevent adverse promitotic and prosurvival pathways triggered by PPAR*γ* and its ligands. This therapeutic approach is assumed to be reasonable in tumors where the tumor-suppressor activities of PPAR*γ* are lost/reduced/dysfunctional and should be restored. However, it may not be applicable for tumors where high PPAR*γ* expression/activity levels positively correlate with the state of malignancy. Since no PPAR*γ* antagonist or PPAR*γ* modulator is in clinical use so far, future studies have to evaluate whether (depending on the tumor type and stage) the combination of the latter drugs with kinase inhibitors may be of therapeutical benefit in tumor entities with high PPAR*γ* expression.

## Figures and Tables

**Table 1:** Combination therapy with PPAR*γ* ligands.

Cancer type	PPAR*γ* ligand	Combination	Inhibitor type	Reference
In vitro				

CML	TZD18	Imatinib	Abl, other RPTKs	[122]
NSCLC A549	Rosiglitazone	Gefitinib	EGFR/Her1	[144]
Breast MCF7	Troglitazone	Herceptin	Mab-Her2/ErbB2	[145]

In vivo (human xenografts or chemically-induced tumors in rodents)				

NSCLC A549	Pioglitazone	PX-866 Gefitinib	PI3K-p110*α* Her1/EGFR	[149]
Breast (by NMU)	Rosiglitazone	Tamoxifen	SERM	[150]
Thyroid ATC	RS5444	Paclitaxel	Chemotherapeutic	[120]

Clinical studies				

Melanoma Sarcoma	Pioglitazone	Rofecoxib Trofosfamide	COX2 Chemotherapeutic	[151–153]
Advanced Solid tumors	LY293111	Irinotecan Gemcitabine	Chemotherapeutic Chemotherapeutic	[154–156]
Glioblastoma Anaplastic Glioma	Pioglitazone	Rofecoxib Capecitabine Temozolomide	COX2 Chemotherapeutic Chemotherapeutic	[157]

## ABBREVIATIONS

AF: Activation function
DBD: DNA binding domain
COX: Cyclooxygenase
ERK: Extracellular signal-regulated kinase
LBD: Ligand binding domain
MAPK: Mitogen-activated protein kinase
MEK: MAPK/ERK kinase
NSAID: Nonsteroidal anti-inflammatory drug
NCoA: NR coactivator
NCoR: NR corepressor
NR: Nuclear receptor
PPAR: Peroxisome proliferator-activated receptor

PPRE: PPAR responsive element
RXR: Retinoid X receptor
ERK cascade: Ras-Raf-MEK1/2-ERK1/2 cascade.
